# Precision of CT-derived alveolar recruitment assessed by human observers and a machine learning algorithm in moderate and severe ARDS

**DOI:** 10.1186/s40635-023-00495-6

**Published:** 2023-02-17

**Authors:** Ludmilla Penarrubia, Aude Verstraete, Maciej Orkisz, Eduardo Davila, Loic Boussel, Hodane Yonis, Mehdi Mezidi, Francois Dhelft, William Danjou, Alwin Bazzani, Florian Sigaud, Sam Bayat, Nicolas Terzi, Mehdi Girard, Laurent Bitker, Emmanuel Roux, Jean-Christophe Richard

**Affiliations:** 1 Université Claude Bernard Lyon 1, INSA-Lyon, CNRS, INSERM, CREATIS UMR 5220, U1294, Université de Lyon, Villeurbanne, France; 2 Service de Médecine Intensive Réanimation, Hôpital de la Croix Rousse, Hospices Civils de Lyon, 103 Grande Rue de La Croix Rousse, 69004 Lyon, France; 3 Service de Radiologie, Hôpital De La Croix Rousse, Hospices Civils de Lyon, Lyon, France; 4 Université de Lyon, Université Claude Bernard Lyon 1, Villeurbanne, France; 5 Service de Médecine-Intensive Réanimation, CHU Grenoble-Alpes, Grenoble, France; 6 Synchrotron Radiation for Biomedicine Laboratory (STROBE), INSERM UA07, Univ. Grenoble Alpes, Grenoble, France; 7 Department of Pulmonology and Physiology, Grenoble University Hospital, Grenoble, France; 8 Maladies Infectieuses et Réanimation Médicale, CHU Rennes, Rennes, France; 9 Faculté de Médecine, Biosit, Université Rennes1, Rennes, France; 10 INSERM-CIC-1414, Faculté de Médecine, IFR 140, Université Rennes I, Rennes, France

**Keywords:** Acute respiratory distress syndrome, Computed tomography, Alveolar recruitment, Bias, Repeatability, Reproducibility, Measurement error, Machine learning

## Abstract

**Background:**

Assessing measurement error in alveolar recruitment on computed tomography (CT) is of paramount importance to select a reliable threshold identifying patients with high potential for alveolar recruitment and to rationalize positive end-expiratory pressure (PEEP) setting in acute respiratory distress syndrome (ARDS). The aim of this study was to assess both intra- and inter-observer smallest real difference (SRD) exceeding measurement error of recruitment using both human and machine learning-made lung segmentation (i.e., delineation) on CT. This single-center observational study was performed on adult ARDS patients. CT were acquired at end-expiration and end-inspiration at the PEEP level selected by clinicians, and at end-expiration at PEEP 5 and 15 cmH_2_O. Two human observers and a machine learning algorithm performed lung segmentation. Recruitment was computed as the weight change of the non-aerated compartment on CT between PEEP 5 and 15 cmH_2_O.

**Results:**

Thirteen patients were included, of whom 11 (85%) presented a severe ARDS. Intra- and inter-observer measurements of recruitment were virtually unbiased, with 95% confidence intervals (CI_95%_) encompassing zero. The intra-observer SRD of recruitment amounted to 3.5 [CI_95%_ 2.4–5.2]% of lung weight. The human–human inter-observer SRD of recruitment was slightly higher amounting to 5.7 [CI_95%_ 4.0–8.0]% of lung weight, as was the human–machine SRD (5.9 [CI_95%_ 4.3–7.8]% of lung weight). Regarding other CT measurements, both intra-observer and inter-observer SRD were close to zero for the CT-measurements focusing on aerated lung (end-expiratory lung volume, hyperinflation), and higher for the CT-measurements relying on accurate segmentation of the non-aerated lung (lung weight, tidal recruitment…). The average symmetric surface distance between lung segmentation masks was significatively lower in intra-observer comparisons (0.8 mm [interquartile range (IQR) 0.6–0.9]) as compared to human–human (1.0 mm [IQR 0.8–1.3] and human–machine inter-observer comparisons (1.1 mm [IQR 0.9–1.3]).

**Conclusions:**

The SRD exceeding intra-observer experimental error in the measurement of alveolar recruitment may be conservatively set to 5% (i.e., the upper value of the CI_95%_). Human–machine and human–human inter-observer measurement errors with CT are of similar magnitude, suggesting that machine learning segmentation algorithms are credible alternative to humans for quantifying alveolar recruitment on CT.

## Background

Quantitative computed tomography (CT) has been extensively used to study acute respiratory distress syndrome (ARDS) morphology and physiopathology and may be used in the near future to guide ARDS treatment [1, 2], but it requires time-consuming manual segmentation of the lungs by trained physicians (i.e., delineation of lung boundaries in order to quantify multiple lung CT parameters). This process is particularly challenging in ARDS patients, as non-aerated lung regions are often poorly or non-distinguishable from the surrounding structures, especially in CT-images acquired without iodine-based contrast materials.

However, repeatability (intra-observer variability) and reproducibility (inter-observer variability among other) of the manual lung segmentation technique have been poorly addressed in both experimental ARDS [3] and clinical acute respiratory failure studies [4], and no studies has addressed to date this issue in ARDS patients, especially in the most severely ill treated with extracorporeal membrane oxygenation (ECMO). The issue of segmentation errors is especially critical for the measurement of alveolar recruitment, as it is computed as the change in weight of the non-aerated compartment (i.e., the one most prone to segmentation errors) between two positive end-expiratory pressure (PEEP) levels [5], and hence is based on a difference between measurements (i.e., combining measurement errors of two consecutive measurements).

Therefore, the smallest real difference (SRD) exceeding measurement error of alveolar recruitment assessed with CT is to date unknown. This can be computed by the repeatability and reproducibility coefficients (assessing intra- and inter-observer variability) which quantify absolute measurement error in the same units as the measurement tool [6], both being required to reliably assess precision of a measurement tool. Assessing measurement error in alveolar recruitment on CT is of paramount importance to select a reliable threshold identifying ARDS patients with high potential for alveolar recruitment and to rationalize PEEP setting.

A modern answer to the issue of repeatability of CT-derived parameters should not be restricted to human–human inter-observer variability, as machine learning (ML) algorithms are increasingly used as automated lung segmentation tools [7–9]. Our hypothesis was that human–ML may be lower than human–human inter-observer variability.

The primary aim of the present study was to assess both intra- and inter-observer SRD of alveolar recruitment detectable on both manually and ML segmented CT of ARDS patients. Secondary aims of the study were:

- to assess intra- and inter-observer SRD measured by both human and ML operators in other commonly used CT-quantitative parameters,
- to assess intra- and inter-observer bias in CT-quantitative parameters measured by human and ML operators,
- to quantify intra- and inter-observer accuracy of lung segmentations.

## Methods

### Study design and setting

This study is a single-center ancillary study of an ongoing prospective observational multicenter study performed in two intensive care units (ICU) located in university hospitals. The study was approved by our institutional ethics committee (CSE HCL20_194). Consecutive patients were enrolled between June 21st, 2021, and September 15th, 2021. Consent for data utilization was sought from the patients or their representative. Some CT data of these patients have been used in a previous study [10].

### Patients

Eligible patients were aged 18 or older, under invasive mechanical ventilation, with ARDS [11] and a ratio of oxygen partial pressure in arterial blood over inspired oxygen fraction (PaO_2_/FiO_2_) below 300 Torr, and had an indication for CT according to their attending physician.

Exclusion criteria were ARDS onset > 72 h or ECMO-onset > 72 h, contra-indication to the transport to the imaging facility (PaO_2_/FIO_2_ < 60 Torr, mean arterial pressure < 65 mmHg, or intracranial hypertension), inability to sustain a 10-s respiratory pause without respiratory effort, presence of intrathoracic metallic devices, previous inclusion in the present study, chronic obstructive pulmonary disease, pneumothorax or broncho-pleural fistula, pregnancy, patients under a legal protective measure and refusal to participate by patient and/or relative.

### Protocol description

The non-ECMO patients received ventilation with tidal volume (VT) 4 to 6 mL.kg^−1^ of predicted body weight (PBW) to keep plateau pressure (P_Plat,rs_) < 30 cmH_2_O [12]. To adjust the PEEP, the ICU policy was to use a PEEP-FiO_2_ table [13]. The ECMO patients underwent ventilation with VT around 1 mL.kg^−1^ PBW and PEEP adjusted to target P_Plat,rs_ around 20 cmH_2_O.

Respiratory measurements and arterial blood gas analysis were performed at inclusion at least 1 h after adjustment of ventilatory settings.

The patients were then transferred to the imaging facility using a MONNAL T60 ventilator (Air Liquide Medical Systems, Antony, France). The endotracheal tube was briefly occluded with a Kocher clamp during ventilator change to avoid derecruitment.

### Data collection

The following variables were recorded at inclusion: anthropometric and demographic data, time of ARDS identification, ARDS severity and risk factors, Simplified Acute Physiology Score 2 (SAPS 2) [14] and SOFA score [15], ventilatory settings, respiratory measurements, and arterial blood gas.

### CT measurements

Low-dose CT acquisitions were performed in supine position with an iCT 256 or Ingenuity CT (Philips Healthcare, Eindhoven, The Netherlands) using the following settings: voltage 140 kVP, slice thickness 1 mm, matrix size 512 × 512. Field of view (FOV), pixel size and tube current–time product were adapted for each patient.

Four different CT acquisitions were performed from apex to base during end-expiratory or end-inspiratory pauses: one end-expiratory and one end-inspiratory CT at the PEEP level set by the attending clinician, an end-expiratory CT at PEEP 15 cmH_2_O, and an end-expiratory CT at PEEP 5 cmH_2_O. The absence of respiratory efforts during the pauses was checked on the ventilator pressure–time curves.

CT images reconstruction was performed using a smooth filter (kernel B).

### Lung segmentation

The lungs were manually segmented by two independent observers with a CreaTools-based software [16], by drawing the external boundaries of the lungs, excluding pleural effusions, main bronchi and main pulmonary arteries from lung region-of-interest. Observer#1 performed two independent lung segmentations on each CT of the whole dataset to assess intra-observer variability, with a time lag between successive segmentations of the same CT amounting to at least 3 months. Observer#2 performed a single segmentation on each CT of the whole dataset to assess human–human inter-observer variability.

The lungs were also segmented with a deep 3D convolutional neural network using a modified version of the 3D U-net architecture [17]. A lightweight version of the 3D U-net was implemented with five convolutional layers (starting with 4 filters and doubling at each layer up to 64 filters before the model bottleneck). The model was trained with 316 CT volumes from 97 patients (yielding a Dice similarity coefficient of 0.972 on the training set); none of which being included in the present study. Since the FOV and the pixel size were adapted for each patient, every volume was preprocessed to obtain an isotropic voxel size of 1 mm, resulting in an image size of 448 × 448 × 320. The model was optimized with the Adam optimizer through a Dice-based loss function [18]. The trained 3D convolutional model performed lung segmentation on each CT of the whole dataset to assess human–ML inter-observer variability [18].

### Computation of CT parameters

Segmented lung volumes were analyzed using MATLAB (MathWorks, Natick, MA). The following CT parameters were assessed on each patient for each set of segmented CTs (i.e., 1st and 2nd set of observer #1 segmentations, observer #2 and ML segmentations).

Voxel tissue and gas fraction were computed as previously described [19]. Tissue and gas volumes were computed as the product of their respective fractions times voxel volume times number of voxels in segmented lung volume.

Lung parenchyma was classified into four compartments, according to CT number: non-aerated (density between + 100 and − 100 Hounsfield units (HU)), poorly aerated (density between − 101 and − 500 HU), normally aerated (density between − 501 and − 900 HU), and hyper-aerated tissue (density ≤ − 901 HU). Total lung weight and weight of each compartment were estimated using lung tissue volume, assuming a tissue density of 1 g.mL^−1^ [20]. The non-aerated compartment weight was standardized to total lung weight, while the aerated volume within the hyper-aerated compartment was standardized to PBW (as tissue weight is negligible in this compartment).

Alveolar recruitment between PEEP 5 and 15 cmH_2_O was computed as the weight of the non-aerated compartment at PEEP 5 cmH_2_O minus its weight at PEEP 15 cmH_2_O and standardized to total lung weight.

Tidal recruitment of the non-aerated compartment was defined as the weight of the non-aerated compartment at end-expiration minus its weight at end-inspiration [21], and standardized to total lung weight.

Tidal hyperinflation was computed as the volume of the hyper-aerated compartment at end-inspiration minus its volume at end-expiration [21], and standardized to PBW.

### Assessment of lung segmentation accuracy

To assess the intra- and inter-observer accuracy of lung segmentations, the following metrics were computed:

- the Dice similarity coefficient [18], a measure of overlap of two lung segmentation masks, computed as
  1 $$\mathrm{DSC}=\frac{2\times |X\cap Y|}{|X|+|Y|},$$

with X and Y being two lung segmentation masks, $|X\cap Y|$ the number of voxels common to both segmentation masks, and $|X|+|Y|$ the total number of voxels in both lung segmentation masks. The DSC ranges from 0 (case of two non overlapping segmentation masks) to 1 in the case of two perfectly identical lung segmentation masks.

- the average symmetric surface distance (ASSD) expressed in mm computed as follows [22]. Surface voxels of two lung segmentation masks were determined as voxels having at least one non-lung voxel within their 26-neighborhood (i.e., adjacent to their 12 edges, 8 corners and 6 faces). For each surface voxel of the first lung segmentation mask, the Euclidean distance to the closest surface voxel of the second lung segmentation mask was computed using the k-NN algorithm [23] and stored. The same process was applied from the surface voxels of the second lung segmentation mask to the closest surface voxel of the first lung segmentation mask in order to provide symmetry, and the ASSD was finally defined as the average of all stored distances, 0 corresponding to a perfect match between the two lung segmentation masks.
- the maximum symmetric surface distance (MSSD) expressed in mm computed as follows [22, 24]. Differences in Euclidean distances between surface voxels of two lung segmentation masks were determined, and the maximum value yielded the MSSD. This measurement is sensitive to outliers and returns the true maximum error.

### Statistical analysis

Statistical analysis was performed using R version 4.1.1 [25] with the following packages multcomp [26], lme4 [27], lmerTest [28], and boot [29, 30]. A *p*-value ≤ 0.05 was chosen for statistical significance.

Data were expressed as count (percentage) or median [interquartile range (IQR)], unless otherwise stated. Between groups comparisons were performed using a linear mixed model, to account for repeated measurements. Multiple comparisons between groups were performed using the Holm–Sidak procedure.

Intra-observer and inter-observer bias and 95% confidence interval (CI_95%_) for the bias were computed for each CT parameters with the Bland and Altman method [31].

The repeatability coefficient (RC), (i.e., the SRD exceeding the measurement error between repeated measurements by the same observer under identical measurement conditions), was computed for each CT parameters as follows [32]:

2 $$\mathrm{RC}={\mathrm{S}}_{\mathrm{W}}\times \sqrt{2}\times 1.96,$$

with S_W_ being the within-subject standard deviation.

The reproducibility coefficient (RDC) (i.e., the SRD exceeding the measurement error between different observers under identical measurement conditions) was computed for each CT parameters as follows [33]:

3 $$\mathrm{RDC}={\mathrm{S}}_{\mathrm{B}}\times \sqrt{2}\times 1.96,$$

with S_B_ being the between-subject standard deviation.

CI_95%_ for both repeatability and reproducibility coefficients were computed for each CT parameters using non-parametric bootstrapping, 1000 replicates and the bias-corrected and accelerated method [34].

Sample size was computed with the aim of assessing the within-subject standard deviation with a precision of at most 40% of the population value at a level of alpha error set to 0.05 [32]. Under these assumptions, a sample size of at least 12 patients would be required and the study population was conservatively set to 13.

## Results

### Characteristics at inclusion

**Fig. 1:**
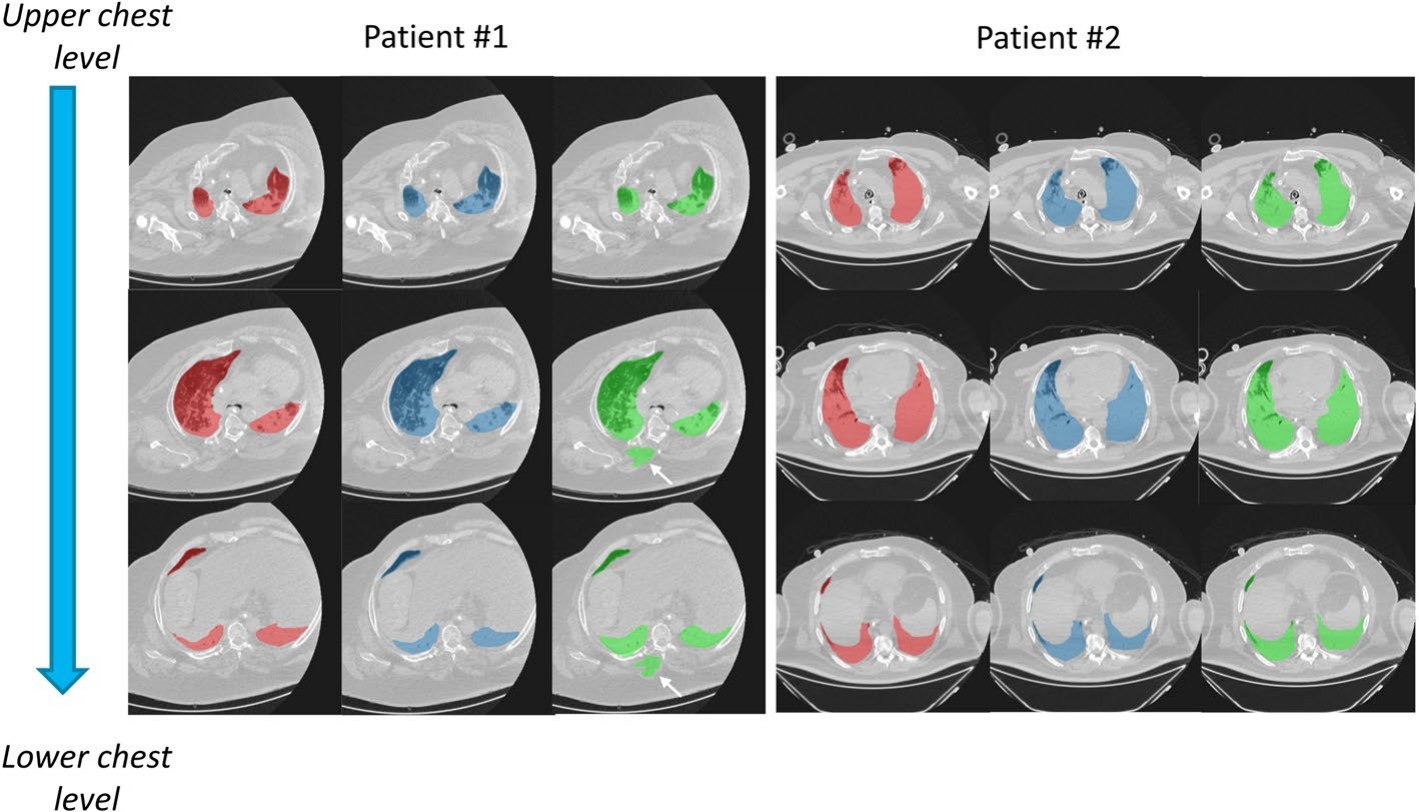
Lung segmentation masks performed by 3 different observers in 2 patients at 3 chest levels. Red regions are lung segmentation masks performed by human operator#1, blue ones are lung segmentation masks performed by human operator#2, and green ones are lung segmentation masks provided by the machine learning algorithm. The arrows denote obvious segmentation errors by the machine learning algorithm

Thirteen patients were included and their clinical characteristics are reported in Table 1. We included mostly severe ARDS [11 patients (85%)] according to the Berlin definition [35], two of whom (15%) being under ECMO, and all patients but one presented COVID-19 related ARDS (92%). CT scans were mostly realized during the first 24 h after ARDS onset. Two CT volumes were missing for one patient, ending up in a total of 50 CT volumes analyzed. Lung segmentation masks provided by human operators and the ML algorithm in two representative patients are presented in Fig. 1.

**Table 1:** Patient characteristics

Variables	Whole dataset (*n* = 13)
Sex male—no. (%)	8 (62%)
Median age [IQR]—year	65 [57–72]
Median BMI [IQR]—kg.m^−2^	29 [24–37]
Median delay between ARDS onset and CT [IQR]—day	1 [0–1]
ARDS severity—no. (%) Moderate Severe without ECMO Severe under ECMO	2 (15%) 9 (70%) 2 (15%)
COVID-19—no. (%)	12 (92%)
Median SAPS 2 at ICU admission [IQR]	42 [38–53]
Median SOFA score at inclusion [IQR]	8 [7–9]
Vasopressor—no. (%)	11 (85%)
Median VT at inclusion [IQR]—mL.kg^−1^ PBW	6.0 [5.9–6.0]
Median PEEP at inclusion [IQR]—cmH_2_O	8 [5–10]
Median PEEP_tot,rs_ at inclusion [IQR]—cmH_2_O	9 [6–10]
Median P_plat,rs_ at inclusion [IQR]—cmH_2_O	20 [18–21]
Median ∆P_rs_ at inclusion [IQR]—cmH_2_O	11 |9–13]
Median pH at inclusion [IQR]	7.36 [7.30–7.39]
Median PaO_2_ at inclusion [IQR]—Torr	70 [62–75]
Median FiO_2_ or FmO_2_ at inclusion [IQR]—% ^†^	100 [90–100]
Median PaCO_2_ at inclusion [IQR]—Torr	47 [40–49]
Day-90 mortality—no. (%)	6 (46%)
Median ventilator-free days at day-60 [IQR]—day	0 [0–38]
Median ICU length of stay [IQR]—day	27 [10–44]

ARDS denotes acute respiratory distress syndrome; BMI, body mass index; CT, computed tomography; ∆P_rs_, driving pressure of the respiratory system; ECMO, extracorporeal membrane oxygenation; FiO_2_ inspired oxygen fraction; FmO_2_, extracorporeal membrane oxygen fraction; ICU, intensive care unit; IQR, interquartile range; PaCO_2_, carbon dioxide partial pressure in arterial blood; PaO_2_, oxygen partial pressure in arterial blood; PBW, predicted body weight; PEEP, positive end-expiratory pressure; PEEP_tot,rs_, total PEEP of the respiratory system; P_plat,rs_, plateau pressure of the respiratory system; SAPS 2 Simplified Acute Physiology Score 2; VT, tidal volume

^†^FiO_2_ is provided for non-ECMO patients, and FmO_2_ for ECMO patients

CT-derived lung measurements are reported in Table 2. Extent of lung injury was high in the study population as shown by a median non-aerated lung at PEEP 5 cmH_2_O amounting to 59 [44–68] % of total lung weight. Median recruitment between PEEP 5 and 15 cmH_2_O amounted to 5.8 [4.2–9.2] % of total lung weight.

**Table 2:** CT-derived lung measurement

Variables	Median [IQR]	Range
Alveolar recruitment PEEP_5–15_—% lung weight	5.8 [4.2–9.2]	− 3.3–21.6
Lung weight at PEEP 5—g	1346 [1152–1681]	747–2343
Lung weight at PEEP 15—g	1347 [1155–1715]	712–2328
Non-aerated lung at PEEP 5—% lung weight	59 [44–68]	25–91
Non-aerated lung at PEEP 15—% lung weight	55 [36–60]	21–86
Tidal hyperinflation—mL.kg^−1^ PBW	0.4 [0.2–1]	0.0–5.2
Tidal recruitment—% lung weight	4.2 [1.9–6.4]	− 6.2–14.3
End-expiratory lung volume at PEEP 5—mL	682 [473–880]	141–2748
End-expiratory lung volume at PEEP 15—mL	970 [789–1354]	251–3365
Hyperinflation at PEEP 5—mL.kg^−1^ PBW	0.1 [0.1–0.4]	0.0–20.3
Hyperinflation at PEEP 15—mL.kg^−1^ PBW	0.4 [0.1–0.7]	0.0–29.7

Values are pooled values from measurements of observer#1 (measurement #1 and #2), observer#2 and machine learning

CT denotes computed tomography; IQR, interquartile range; PEEP, positive end-expiratory pressure; PEEP_5–15_, PEEP change from 5 to 15 cmH_2_O; and PBW predicted body weight

### Assessment of lung segmentation accuracy (Fig. 2)

**Fig. 2:**
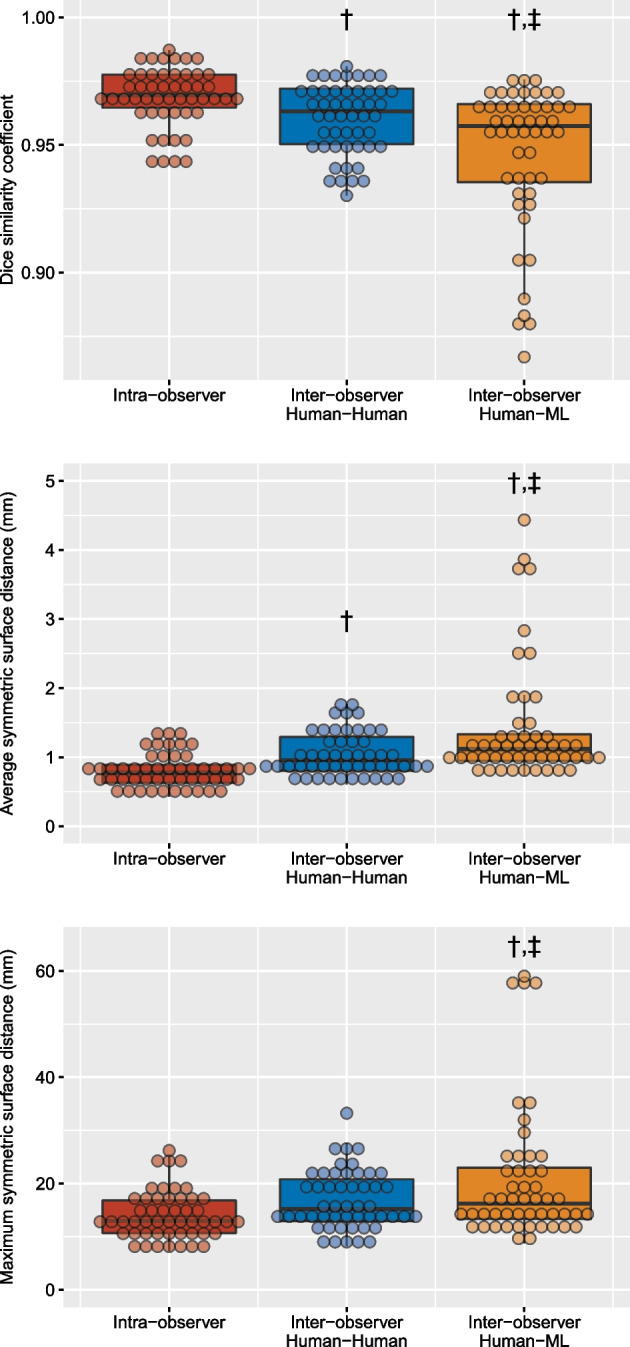
Accuracy of lung segmentation in various conditions (intra-observer, inter-observer human–human and inter-observer human–ML). Each point refers to one computed tomography volume acquisition (four computed tomography volumes were acquired for each patient, i.e., at both end-expiration and end-inspiration at the PEEP level chosen by attending physician, and at end-expiration at PEEP 5 and 15 cmH_2_O). ^†^*p* < 0.05 vs intra-observer, ^‡^*p* < 0.05 vs inter-observer (human–human). ML denotes machine learning and PEEP positive end-expiratory pressure

The Dice similarity coefficient, gauging the overlap of two segmentations masks, was close to 1, for intra-observer comparisons (0.97 [0.96–0.98]), human–human inter-observer comparisons (0.96 [0.95–0.97]) or human–ML inter-observer comparisons (0.96 [0.94–0.97]), but was however significantly lower in the latter group (Fig. 2).

The ASSD (i.e., the average difference between the surface of 2 segmentation masks in three dimensions) was significantly lower for intra-observer comparisons (0.8 [0.6–0.9] mm) as compared to both human–human inter-observer comparisons (1.0 [0.8–1.3] mm) and human–ML inter-observer comparisons (1.1 [0.9–1.3] mm) (Fig. 2).

The MSSD (i.e., the highest distance between the surface of two segmentation masks in three dimensions) was not significantly different between the intra-observer comparisons (12.9 [10.7–16.8] mm) and human–human inter-observer comparisons (15.2 [12.9–20.8] mm), but was significantly higher in the human–ML inter-observer comparisons (16.2 [13.3–23.0] mm) (Fig. 2).

The four individual points behaving as outliers in the human–ML comparisons (Fig. 2) with respect to the three above-mentioned metrics correspond to the 4 CT of a single patient with obvious segmentation errors by the ML algorithm (i.e., patient #1 in Fig. 1).

### Bias

**Fig. 3:**
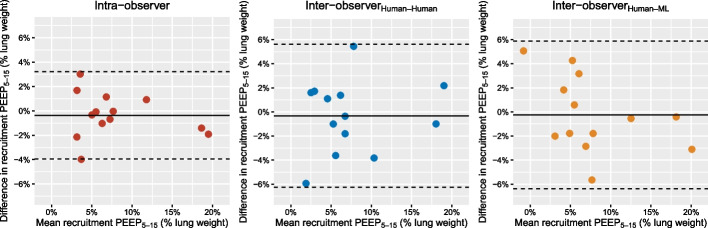
Repeatability of alveolar recruitment measurements assessed using Bland and Altman plots in the intra-observer, human–human inter-observer and human–ML inter-observer settings. Each datapoint refers to individual measurements. Continuous lines are mean bias in each repeatability or reproducibility conditions. Broken lines refer to lower and upper limits of agreements in each repeatability or reproducibility conditions. ML denotes machine learning, and recruitment PEEP_5-15_ alveolar recruitment by PEEP increase from 5 to 15 cmH_2_O

The intra- and inter-observer measurements of alveolar recruitment were virtually unbiased, with CI_95%_ encompassing zero in all repeatability or reproducibility conditions (Fig. 3, Table 3). As for the other parameters exposed in Table 3, there was no significant intra- and inter-observer bias apart for lung weight and end-expiratory lung volume, as shown by their CI_95%_ excluding zero, but the magnitude of the bias was very low.

**Table 3:** Intra-observer and inter-observer bias for computed tomography measurements

Variables	Intra-observer bias	Inter-observer bias (human–human)	Inter-observer bias (human–ML)
Alveolar recruitment PEEP_5–15_—% lung weight	− 0.4 [− 1.5–0.7]	− 0.3 [− 2.1–1.5]	− 0.3 [− 2.1–1.6]
Lung weight at PEEP 5—g	− 41 [− 70 to − 12]	85 [48–122]	34 [− 28–97]
Lung weight at PEEP 15—g	− 30 [− 60 to − 1]	89 [53–126]	29 [− 34–91]
Non-aerated lung at PEEP 5—% lung weight	− 0.7 [− 1.8–0.2]	1.0 [− 0.4–2.4]	0.7 [− 1.6–3.0]
Non-aerated lung at PEEP 15—% lung weight	− 0.4 [− 1.6–0.7]	1.3 [0.2–2.3]	1.0 [− 1.8–3.7]
Tidal hyperinflation—mL.kg^−1^ PBW	0.00 [− 0.01–0.01]	0.01 [0.00–0.02]	0.03 [− 0.04–0.09]
Tidal recruitment—% lung weight	0.6 [− 1.0–2.2]	-0.5 [− 2.4–1.3]	− 0.8 [− 2.3–0.8]
End-expiratory lung volume at PEEP 5—mL	− 1 [− 4–2]	7 [4–10]	4 [− 4–11]
End-expiratory lung volume at PEEP 15—mL	0 [6–7]	9 [5–13]	6 [− 7–19]
Hyperinflation at PEEP 5—mL.kg^−1^ PBW	0.00 [− 0.01–0.01]	0.01 [0.00–0.03]	0.01 [− 0.01–0.03]
Hyperinflation at PEEP 15—mL.kg^−1^ PBW	0.00 [− 0.02–0.01]	0.02 [0.00–0.04]	0.03 [− 0.03–0.10]

Values are mean [CI_95%_]

CI_95%_ denotes 95% confidence interval; ML, machine learning; PEEP, positive end-expiratory pressure; PEEP_5–15_, PEEP change from 5 to 15 cmH_2_O; and PBW predicted body weight

### Repeatability and reproducibility coefficient

The repeatability coefficient (or intra-observer SRD) of alveolar recruitment measurement amounted to 3.5% [CI_95%,_ 2.4–5.2] % of total lung weight, and the upper value of this CI_95%_ would have identified 8 (62%) patients as being above the measurement error (and hence considered as presenting significant alveolar recruitment). The human–human reproducibility coefficient (or inter-observer SRD) was slightly higher amounting to 5.7 [CI_95%_ 4.0–8.0] % of total lung weight, as was the human–ML reproducibility coefficient (5.9 [CI_95%_ 4.3–7.8] % of total lung weight). In the other measures reported in Table 4, both repeatability and reproducibility coefficients (human–human and human–ML) were close to zero for the CT-measurements focusing on aerated lung (end-expiratory lung volume, hyperinflation), and higher for the CT-measurements relying on accurate segmentation of the non-aerated lung (lung weight, tidal recruitment, non-aerated lung).

**Table 4:** Repeatability and reproducibility coefficients for computed tomography measurements

Variables	Intra-observer RC	Inter-observer RDC (human–human)	Inter-observer RDC (human–ML)
Alveolar recruitment PEEP_5–15_—% lung weight	3.5 [2.4–5.2]	5.7 [4.0–8.0]	5.9 [4.3–7.8]
Lung weight at PEEP 5—g	121 [78–181]	202 [146–300]	207 [139–270]
Lung weight at PEEP 15—g	111 [79–189]	209 [152–278]	203 [143–274]
Non-aerated lung at PEEP 5—% lung weight	3.5 [2.6–4.5]	4.7 [3.7–6.3]	7.4 [5.3–10.3]
Non-aerated lung at PEEP 15—% lung weight	3.6 [1.9–6.4]	4.2 [2.0–6.9]	8.8 [5.0–12.9]
Tidal hyperinflation—mL.kg^−1^ PBW	0.02 [0.01–0.03]	0.03 [0.02–0.04]	0.2 [0.01–0.45]
Tidal recruitment—% lung weight	4.9 [2.7–8.8]	5.6 [3.7–8.2]	4.8 [2.9–6.9]
End-expiratory lung volume at PEEP 5—mL	10 [7–15]	17 [11–26]	24 [7–51]
End-expiratory lung volume at PEEP 15—mL	21 [7–43]	22 [14–34]	41 [9–91]
Hyperinflation at PEEP 5—mL.kg^−1^ PBW	0.03 [0.02–0.04]	0.05 [0.02–0.09]	0.06 [0.01–0.14]
Hyperinflation at PEEP 15—mL.kg^−1^ PBW	0.04 [0.02–0.07]	0.08 [0.03–0.13]	0.22 [0.01–0.50]

Values are mean [CI_95%_]

CI_95%_ denotes 95% confidence interval; ML, machine learning; PEEP, positive end-expiratory pressure; PEEP_5–15_, PEEP change from 5 to 15 cmH_2_O; PBW, predicted body weight; RC, repeatability coefficient; and RDC reproducibility coefficient

## Discussion

The main findings of the study were that: (1) the smallest real difference in alveolar recruitment exceeding CT-measurement error may be conservatively set to 5% (i.e., the upper value of the CI_95%_ in the repeatability condition); (2) human–ML and human–human inter-observer variability of alveolar recruitment measurement by CT are of similar magnitude, suggesting that ML segmentation algorithms are credible alternative to human operators for quantifying alveolar recruitment on CT; (3) inter-observer repeatability and reproducibility of other CT measurements are acceptable, even for measurements relying on accurate segmentation of the non-aerated lung.

The present study was performed in a population characterized by its severity (85% of severe ARDS, 15% under ECMO, median [IQR] non-aerated lung at PEEP 5 cmH_2_O amounting to 59 [44–68]% of total lung weight). For comparison, non-aerated lung at PEEP 5 cmH_2_O amounted to 37% of total lung weight in the seminal study by Gattinoni on non-COVID ARDS [5]. Since the reliability of manual segmentation is expected to be inversely related to the extent of lung non-aerated compartment, our results should apply to the whole range of ARDS severity.

The measure of alveolar recruitment is fundamental to identify responders to higher PEEP levels during ARDS. Visual semi-quantitative assessment of lung recruitability has been proposed [36], but the precision of the measurement was poor (i.e., ± 7% of total lung weight). On the other hand, quantitative measurement of alveolar recruitment using CT requires to correctly identify non-aerated pulmonary areas during the manual lung segmentation process. The magnitude reported for the inter-observer variability of manual segmentation is approximately 2% of the region-of-interest volume in previous studies [3, 4, 37]. Even though manual lung segmentation has been used to evaluate recruitment on CT since the mid-1980s, our study is, to our knowledge, the first assessing the absolute measurement error in alveolar recruitment quantified on CT. Given the lack of widely accepted threshold to identify lung recruitability, low and high recruiters were often separated, in previous studies, on the basis of quartiles values of the population [5, 38, 39] (i.e., a strategy highly dependent on the population case-mix regarding lung recruitability). In our study, we showed that 5% of the total lung weight was the upper limit of the measurement error of alveolar recruitment in the intra-observer condition. As manual segmentation of the two CT required to compute alveolar recruitment is performed by a single operator, this value may be viewed as the lowest value that could be used to identify patients with higher potential for recruitment by PEEP.

Contrary to our hypothesis, human–ML reproducibility of recruitment measurement was not lower than human–human reproducibility. Several explanations may be raised to explain this finding. First, training of the ML algorithm was performed on only 97 patients, an amount that may be insufficient to capture the diversity of lung injury and chest shape in ARDS patients, and increasing the size of the training set may improve future versions of the ML algorithm. Second, training of the ML algorithm was performed using manual segmentations as a gold standard, and inaccuracy of manual segmentations may limit the ability of ML algorithm to perform optimally. To our knowledge, the SRD in alveolar recruitment was not assessed in previous studies using ML-based segmentation on ARDS lungs [8, 9]. However, the Dice similarity coefficient and ASSD were similar in our study and in a study by Maiello et al., although performed on less severe ARDS patients as shown by substantially lower extent of the non-aerated compartment in their study [8]. Precision of ML-based measurements was similar in our study and in another study performed on ARDS patients in which limits of agreement between ML and manual segmentation-based recruitment spanned from − 5.5 and + 6.2% of lung weight [9], suggesting that our results may apply to the current generation of ML-based segmentation algorithms.

Regarding the lower repeatability of other CT measurements relying on accurate segmentation of the non-aerated lung (i.e., lung weight, tidal recruitment, non-aerated lung), this finding is not surprising and was previously identified with another ML segmentation algorithm [9].

Some limitations of the present study should be acknowledged. First, the sample size was relatively small, hence limiting the study ability to narrow the estimation of the SRD. However, the 5% upper limit of the CI_95%_ SRD in the intra-observer condition would have identified more than 50% of the patients as recruiters by PEEP in the largest CT study performed on COVID-19 ARDS patients [10], and the physiological implication of lower values (i.e., below 5%) may be questionable. Second, as this study was performed on all but one COVID-19 patients, the generalization of its results to patients with other ARDS risk factors may be questionable, although infectious pneumonia are by far the most frequent ARDS risk factor [40]. Third, different ML algorithms may have different performance, but the present study may be viewed as a proof of concept on their ability to provide reliable estimation of alveolar recruitment on CT in ARDS patients. Finally, model training in ML depends on the reliability of manual segmentations provided as gold standard, and imprecision in manual delineation of the lung in some difficult to delineate areas (such as hilum or juxta diaphragmatic area) could degrade ML performance.

The clinical implications of this study are the following. First, the 5% recruitment value could be used as the lowest threshold candidate to classify patients as potential recruiter by PEEP, and future studies should assess both the physiological meaning and relevance of this threshold. Second, according to our results, the ML technique presents similar reproducibility with human–human inter-observer variability, which may have important implication for ARDS management in the near future. Indeed, using the methodology of the present study, real-time analysis of CT images may be achievable. To date, lung recruitment quantification requires 3–4 h of manual segmentation to analyze the two CT volumes (i.e., PEEP 5 and 15 cm H_2_O) with millimetric slice thickness, while ML-based segmentation of the two CT volumes was achieved in less than 5 min in the present study. This may be a first step toward the development of new strategies aiming to personalize ARDS ventilation using imaging (e.g., quasi-instantaneous lung segmentation with ML to provide ‘real-time’ measurement of alveolar recruitment helping to personalize PEEP setting [1]).

## Conclusions

The manual lung segmentation technique is repeatable and reproducible in the evaluation of alveolar recruitment by PEEP in ARDS patients, and the smallest real difference exceeding experimental error may be conservatively set to 5%. Human–ML and human–human inter-observer measurement errors of alveolar recruitment by CT are of similar magnitude, suggesting that ML segmentation algorithms are credible alternative to humans for quantifying alveolar recruitment on CT.

## Data Availability

The datasets used and/or analyzed during the current study are available from the corresponding author on reasonable request.

## Abbreviations

ARDS: Acute distress respiratory syndrome
ASSD: Average symmetric surface distance
CI_95%_: 95% Confidence interval
CT: Computed tomography
DSC: Dice similarity coefficient
ECMO: Extracorporeal membrane oxygenation
FiO_2_: Inspired oxygen fraction
FOV: Field of view
HU: Hounsfield unit
ICU: Intensive care unit
IQR: Interquartile range
ML: Machine learning
MSSD: Maximum symmetric surface distance
PaO_2_: Oxygen partial pressure in arterial blood
PBW: Predicted body weight
PEEP: Positive end-expiratory pressure
P_Plat,rs_: Plateau pressure
RC: Repeatability coefficient
RDC: Reproducibility coefficient
SAPS 2: Simplified Acute Physiology Score 2
S_B_: Between-subject standard deviation
SRD: Smallest real difference
S_W_: Within-subject standard deviation
VT: Tidal volume
